# Brain network hierarchy reorganization in subthreshold depression

**DOI:** 10.1016/j.nicl.2024.103594

**Published:** 2024-03-18

**Authors:** Xiaolong Yin, Junchao Yang, Qing Xiang, Lixin Peng, Jian Song, Shengxiang Liang, Jingsong Wu

**Affiliations:** a National-Local Joint Engineering Research Center of Rehabilitation Medicine Technology, Fujian University of Traditional Chinese Medicine, Fuzhou 350122, China; b Rehabilitation Industry Institute, Fujian University of Traditional Chinese Medicine, Fuzhou 350122, China; c College of Rehabilitation Medicine, Fujian University of Traditional Chinese Medicine, Fuzhou 350122, China; d Traditional Chinese Medicine Rehabilitation Research Center of State Administration of Traditional Chinese Medicine, Fujian University of Traditional Chinese Medicine, Fuzhou 350122, China

**Keywords:** Subthreshold depression, Gradient, Default mode network, Hierarchy

## Abstract

**Background:**

Hierarchy is the organizing principle of human brain network. How network hierarchy changes in subthreshold depression (StD) is unclear. The aim of this study was to investigate the altered brain network hierarchy and its clinical significance in patients with StD.

**Methods:**

A total of 43 patients with StD and 43 healthy controls matched for age, gender and years of education participated in this study. Alterations in the hierarchy of StD brain networks were depicted by connectome gradient analysis. We assessed changes in network hierarchy by comparing gradient scores in each network in patients with StD and healthy controls. The study compared different brain subdivisions if there was a different network. Finally, we analysed the relationship between the altered gradient scores and clinical characteristics.

**Results:**

Patients with StD had contracted network hierarchy and suppressed cortical range gradients. In the principal gradient, the gradient scores of default mode network were significantly reduced in patients with StD compared to controls. In the default network, the subdivisions of reduced gradient scores were mainly located in the precuneus, superior temporal gyrus, and anterior and posterior cingulate gyrus. Reduced gradient scores in the default mode network, the anterior and posterior cingulate gyrus were correlated with severity of depression.

**Conclusions:**

The network hierarchy of the StD changed and was significantly correlated with depressive symptoms and severity. These results provided new insights into further understanding of the neural mechanisms of StD.

## Highlights

• Subthreshold depression (StD) patients had contracted network hierarchy and suppressed cortical range gradients.
• In the principal gradient, the gradient scores of default mode network were significantly reduced in StD patients compared to healthy controls.
• The reduced gradient scores in the default mode network, anterior/posterior cingulate gyrus were associated with the severity of depression.
• The network hierarchy changes in StD patients were significantly associated with the severity of depression, providing new insights into the neural mechanisms of StD.

## 1 Introduction

Subthreshold depression (StD) is characterized by the presence of two or more depressive symptoms for at least two weeks, accompanying with some degree of social impairment, but does not meet the diagnostic criteria for major depressive disorder as defined in the Diagnostic and Statistical Manual of Mental Disorders-5 (DSM-5) (Judd et al., 1994). A systematic review of 19 studies estimated the prevalence of StD in adults ranged from 3 % to 10 % in clinical practice and from 1 % to 17 % in community settings (Rodríguez et al., 2012). StD can lead to decreased quality of life and social skills, increased functional impairment and suicide risk (Cuijpers et al., 2004, Balázs et al., 2013, Gilbody et al., 2017, Karsten et al., 2013). Despite the high prevalence and the significant risks of StD, the neurobiological mechanisms underlying it are not yet fully understood.

Functional magnetic resonance imaging (fMRI) is an imaging modality that reflects the level of neural activity in the brain by monitoring changes in the concentration of blood oxygen levels in local areas of the brain, and is one of the effective tools for studying neurobiological mechanisms (Buchbinder, 2016, Wang et al., 2024). It has been found that the human cerebral cortex is organised into several macroscale functional networks, such as the sensorimotor network, the visual network, the limbic network and the default mode network (DMN) (Buckner and Krienen, 2013). In recent years, fMRI studies identified abnormalities in several macroscale functional networks in depression (Kaiser et al., 2015, Li et al., 2018, Yang et al., 2021), and the abnormalities mainly involved the DMN, ventral attention network and limbic network, which were related to emotion processing and the manifestation of several symptoms of depression (Riedel et al., 2018, Luo et al., 2021, Manoliu et al., 2014, Pannekoek et al., 2014). However, there were few studies on macroscale functional networks in StD which was a pre-stage of depression. Thus, there was an urgent need to summarize studies investigating the alterations in the macroscale functional network alterations in StD and to explore potential clinical significance.

Hierarchy is one of the fundamental principles organizing the human brain, enabling the encoding and integration of information from sensory input to cognitive processing (Mesulam, 1998). Advances in classical neuroanatomy and brain imaging techniques have provided the necessary support for the emergence of a functional network hierarchy in the brain. It has been found that the primary sensory network is located at the bottom of the network hierarchy while the DMN is positioned at the top of the hierarchy. And the intermediate network is situated in the middle position of the hierarchy. In addition, the nodes in the core brain regions of the DMN have the longest distance from the primary sensory network that directly controls perception and behaviour (Margulies et al., 2016). Network hierarchy facilitates the implementation of abstract, higher-level brain functions by segregating information that reflects the processing of the instant environment from more self-generated operations in high-level integrative networks (Murphy et al., 2018).

Recent research found that macroscopic brain features could be mapped to a low-dimensional multidimensional representation which was described as a gradient (Vos de Wael et al., 2020). This gradient serves as the axis of cortical features, and regions with similar features tend to occupy similar positions along the cortical gradient axis. Moreover, the study found that one end of the principal gradient of macroscopic cortical organization was anchored to the sensorimotor network while the other end was linked to the DMN, which was consistent with recent descriptions of cortical network gradients (Margulies et al., 2016, Huntenburg et al., 2018). As research on gradients continues, increasing neurological disorders such as Alzheimer's disease, autism spectrum disorders, stroke, major depressive disorder (MDD) and schizophrenia, were considered to have abnormalities in functional network gradients (Hu et al., 2022, Hong et al., 2019, Bayrak et al., 2019, Xia et al., 2022, Dong et al., 2020, Zarkali et al., 2021).

The aim of this study was to investigate whether there were alterations in the gradient network hierarchy in patients with StD compared to healthy controls, and to explore the potential clinical significance of these alterations. We first analysed the the network hierarchy gradient in both StD patients and healthy controls using connectome gradient approach, and examined the changes in the functional network level in the two groups. Finally, we investigated the subdivisions of differential functional network and analyze their clinical significance.

## 2 Methods

### 2.1 Participants

The study was approved and authorized by the Ethics Committee of the Affiliated Rehabilitation Hospital of Fujian University of Traditional Chinese Medicine (approval number: 2019KY-003–01). All participants voluntarily participated in this trial and signed the informed consent.

All the participants were recruited in Fuzhou of China, and were grouped into 43 pairs matched for age, gender and years of education. The diagnostic criteria for the StD group referred to Judd et al.'sdiagnostic criteria (Judd et al., 1994, Rodríguez et al., 2012) including: (1) individuals presented two or more depressive symptoms for most of the day and for at least two weeks (2) they were accompanied by some social impairments and (3) they failed to meet the diagnostic criteria for MDD. Individuals were eligible for enrollment in StD group when they: (1) met the diagnostic criteria of StD (2) had a Center for Epidemiologic Studies Depression (CESD) scores of at least 16 (3) were conscious (4) had no treatment for depression in the past 6 months and (5) had no contraindication to MRI examination. (6) were right handedness. (7) and signed the written informed consent. Individuals were excluded when they: (1) met the diagnostic criteria for mood disorders, bipolar depressive disorder, and psychosis according to the DSM-5 diagnostic criteria (2) had a history of MDD in the last 6 months and a history of head trauma, any significant medical, neurological or psychological illness, (3) had pregnancy, severe hyperthermia or claustrophobia (4) used psychiatric drugs, glucocorticoid drugs, alcohol abuse or addiction disorders (5) participated in other clinical trials that might affect the results of this study (6) were implanted with pacemakers, neurostimulators, intracorporeal microinfusion pumps, artificial heart valves, paramagnetic vascular clips like aneurysmal haemostatic clips, intraocular metal objects, metal/prosthetic implants in the inner ear, metal prostheses/joints, and ferromagnetic foreign bodies (7) suffered from endocrine and metabolic disorders (Cushing's syndrome, Addison's disease, etc.).

### 2.2 Scale assessment information

The CESD scale (Radloff, 1977) is designed to measure the frequency of symptoms associated with depression that subjects have experienced in the past week. It is a sequential scale containing 4 levels: little or no time (<1 day); some or a little time (1–2 days); occasional or moderate time (3–4 days); and most or all of the time (5–7 days). The scores range from 0 to 60, and a score of 16 or higher indicates the presence of depressive symptoms in the individual. Higher scores indicate greater depressive symptoms. The Hamilton Depression (HAMD) scale (Hwang et al., 2016) is a widely used scale in clinical assessment of depression, and consists of 17 items evaluating functions across five domains: anxiety/somatization, retardation, cognitive impairment, sleep disturbance and weight change. Higher scores indicate more severe depressive symptoms.

### 2.3 MRI acquisition

All subjects underwent MRI scans at the Imaging Department of the Affiliated Rehabilitation Hospital of Fujian University of Chinese Medicine, and the resting-state fMRI images were acquired using a Siemens Prisma 3.0T MRI machine and a 64-channel combined head and neck coil. The resting-state fMRI scan sequence was performed using Gradient echo EPI, axial scan, TR/TE/FOV = 2000 ms/30 ms/220 mm*220 mm, flip angle (FA) = 90 degrees, number of layers = 37, layer thickness = 3.5 mm, imaging matrix = 64*64, time points = 180.

### 2.4 MRI preprocessing

Matrix Laboratory (MATLAB 2018b, MathWorks, USA) and Data Processing & Analysis for Brain Imaging (DPABI V7.0, https://rfmri.org/dpabi) software were used for image preprocessing (Yan et al., 2016). The preprocessing steps were adopted from the procedure described by Hu et al (Hu et al., 2022) and were listed as following: (1) removal of the first 5 time points, (2) slice-time correction, (3) realignment, (4) spatial normalisation of the images to a standard EPI template, and resampling the images to 4 mm × 4 mm × 4 mm cubic voxels, (5) spatial smoothing of the data using a Gaussian filter with a full-width at half-maximum of 6 mm × 6 mm × 6 mm, (6) detrending, (7) nuisance covariates regression, including brain white matter signals, global mean signals and cerebrospinal fluid signals, (8) filtering using a band-pass filter (0.01–0.1 Hz), (9) extracting time series for each subject using a atlas of 200 ROIs based on the Schaefer 7 network, which is a commonly used functional network atlas (Schaefer et al., 2018, Yeo et al., 2011), (10) calculating the connectivity matrix using Pearson correlation and subjecting the connectivity matrix to Fisher Z-transformation to make the data more consistent with normal distribution.

### 2.5 Gradient analysis steps

We obtained the scores of the first gradients for each subject using the method described by Wael et al (Vos de Wael et al., 2020). After preprocessing, the Fisher Z-transformed connectivity matrix for each subject was used to calculate the group-averaged connectivity matrix. The connectivity matrix was calculated using BrainSpace Toolbox (http://github.com/MICA-MNI/BrainSpace) (Vos de Wael et al., 2020) to extract 2 group-level gradients from the group-averaged connectivity matrix (dimension reduction technique = diffusion embedding, kernel = cosine similarity matrix, sparsity = 0.9). Group-level gradients were aligned to a subsample of the HCP dataset (N = 217, 122 women, mean ± standard deviation (SD), Age = 28.5 ± 3.7 years) (Vos de Wael et al., 2018) using Procrustes rotation. We calculated the mean of the corresponding Parcels gradient scores for each subject's functional network to represent the gradient scores for each of the seven functional networks (Gonzalez Alam et al., 2022).

### 2.6 Statistical analysis methods

SPSS 26.0 statistical software was used for data analysis. If the measurement data were normally distributed, they were reported as (mean ± SD), and two independent samples *t*-test was used for comparison between groups. If the measurement data were not normally distributed, they were expressed as M(P25 ∼ P75), and Mann-Whitney *U* test was used for comparison between groups. The χ2 test was used for statistical data. *P* < 0.05 was considered a statistically significant difference. Pearson correlations were employed to assess the association between the principal gradient scores of the differential functional networks, and to examine the relationship between differential subdivisions and scores of CESD as well as HAMD.

## 3 Results

### 3.1 Comparison of clinical information and demographic data

There were no significant differences in age, gender and years of education between the control and StD group (*P* > 0.05). However, the StD group had significantly higher scores on the CESD and HAMD scales compared to the control group (*P* < 0.05) (Table 1).

**Table 1:** Clinical and demographics information.

Control(n = 43) Subthreshold depression(n = 43) Z/χ^2^*P*
Age(Year) 19(18 ∼ 22) 19(19 ∼ 20) −0.963 0.336
Sex(M/F)^a^ 16/27 16/27 0.000 1.000
Education(Year) 13(13 ∼ 15) 14(13 ∼ 14) −1.106 0.269
CESD 6(3 ∼ 11) 19(17 ∼ 23) −7.998 < 0.001
HAMD 3(0 ∼ 6) 9(7 ∼ 11) −6.079 < 0.001

Data are presented as the Median (The first quartile ∼ The upper quartile).

Other *P* value obtained by a Mann-Whitney *U* test.

a meant *P* value obtained by a Chi-square test.

### 3.2 Selection of study gradients

**Fig. 1:**
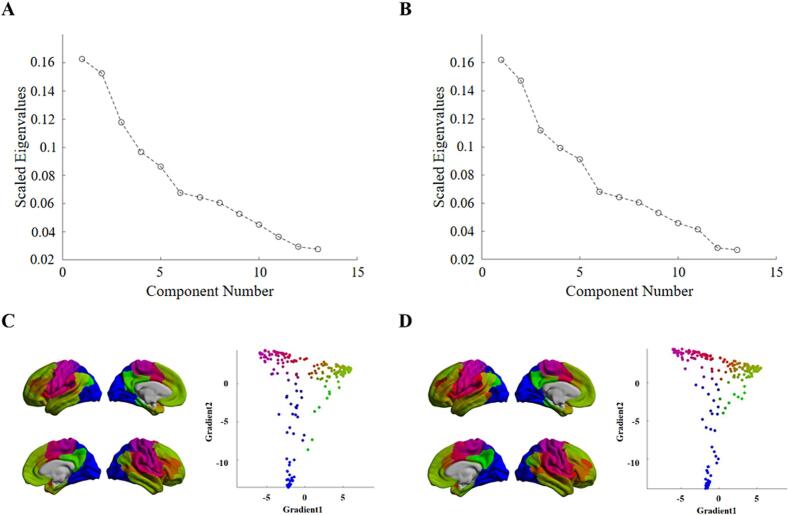
13 eigenvalues of control (A) and StD (B) group.The higher eigenvalues indicates greater importance. Scatter plot of the first two gradients in Control (C) and StD (D) group. Red color represents the sensorimotor network. Blue color represents the visual network. Green color represents the default network. The anchor of the principal gradient is located between the sensorimotor network and the default network, and the anchor of the secondary gradient is situated between the visual network and the sensorimotor network.

In accordance with previous studies, we focused on the eigenvalues of the first 13 gradients, with the higher eigenvalues being more important (Fig. 1. A and B). The eigenvalues tended to decrease, with the principal gradients having the highest eigenvalues. The principle gradients were able to make a trade-off between retaining few components and retaining a large amount of variance (Vos de Wael et al., 2020). The scatter plot depicted the distribution pattern of the principal and secondary gradients (Fig. 1. C and D).In addition, the principal gradients could capture the spatial layout of the cortical hierarchy of the brain (Margulies et al., 2016). Therefore, this study mainly focused on the principal gradients.

### 3.3 Distribution patterns of the principal in the StD group and control group

**Fig. 2:**
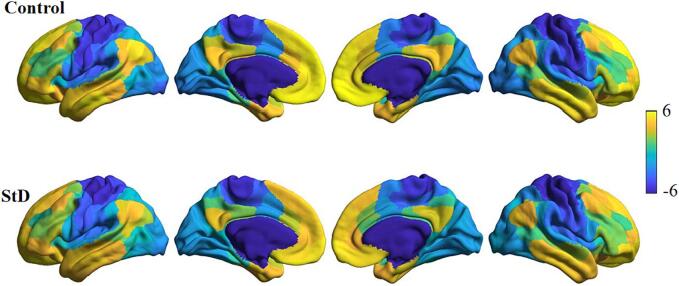
Principal gradient based on Schaefer atlas. In both the control group and the StD group, the principal gradient is positioned between the sensorimotor network and the DMN, with intermediary network regions occupying the middle position.

The result indicated that the principal gradients in both groups displayed a continuum of connectivity variations, ranging from a low level sensorimotor network to a high level DMN, with an intermediate network located in the middle position (Fig. 2).

### 3.4 Comparison of gradients in network levels in both groups and association of gradients with clinical scales

**Fig. 3:**
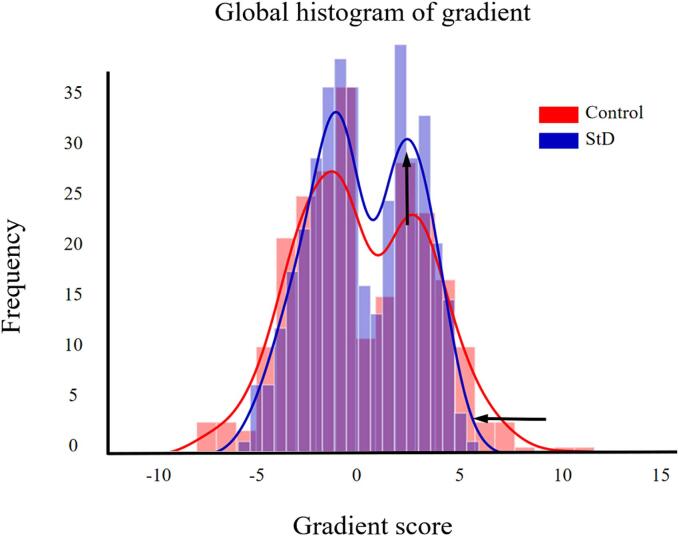
Global histogram of the principal gradient. In the StD group, there is a suppression of extreme values and an increase in values in the mid-range compared to the control group.

**Fig. 4:**
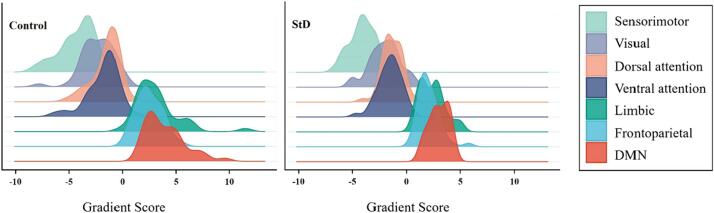
Joy plot of principal gradient based on Yeo 7 functional networks. Compared with the control group, the seven functional networks show a shrinking trend, and the DMN is the most obvious.

**Fig. 5:**
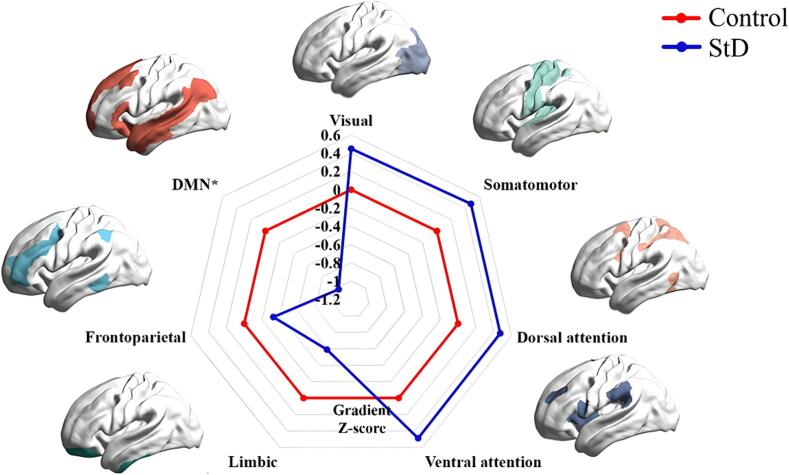
Radar plot of principal gradient based on Yeo 7 functional networks. Compared with the control group, the gradient scores of sensorimotor network, visual network, ventral attention network and dorsal attention network increase, and those of the limbic network, frontoparietal control network and DMN are reduced in the StD group. The gradient scores of DMN are reduced with a significant difference(* *P* < 0.05).

**Fig. 6:**
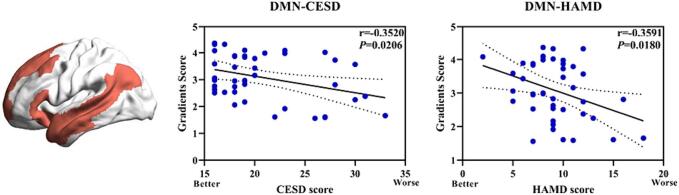
Correlations between altered DMN principal gradient scores and depression scale. The principal gradient scores of DMN are negatively correlated with the scores of CESD and HAMD (*P* < 0.05).

The frequency histograms showed a contraction in the range of the two extremes (i.e., sensorimotor and DMN) and an increase in the medial axis in StD group compared to the control group in the principal gradient (Fig. 3, Fig. 4). Specifically, the gradient scores of the sensorimotor, visual, dorsal attention, and ventral attention networks increased, while those of the limbic, frontoparietal control, and DMN were reduced in the StD group compared to the control group. The gradient scores of DMN were significantly reduced in the StD group compared to the control group, with a statistically difference (*P* < 0.05) (Fig. 5). In StD group, the gradient scores of DMN showed a negative correlation with scale scores of CESD and HAMD (Fig. 6).

### 3.5 Comparison of gradients in brain subdivisions in both groups and association of gradients clinical scales

**Fig. 7:**
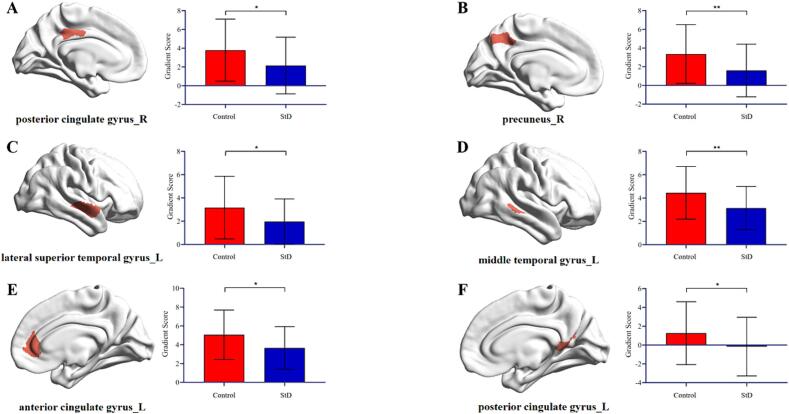
Principal gradient DMN different brain subdivisions plot. The difference in the DMN subdivisions of the principal gradient between the control and StD group (*P < 0.05, **P < 0.01).

**Fig. 8:**
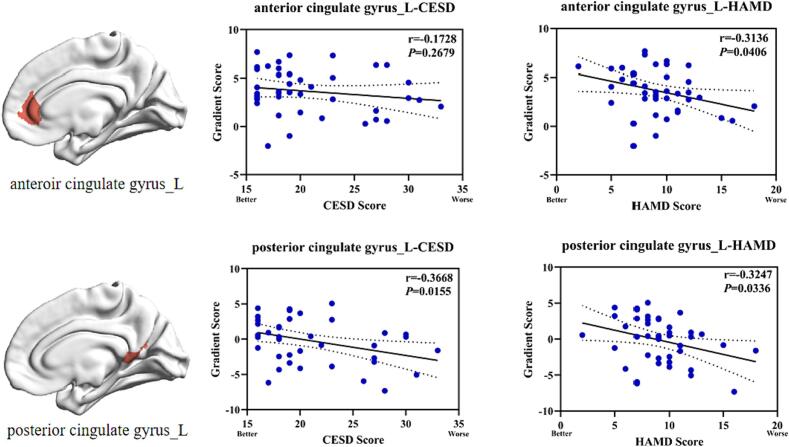
Correlations between altered principal gradient scores of DMN subdivisions and depression scales. The principal gradient scores of left anteroir cingulate gyrus are negatively correlated with the scores of HAMD(*P* < 0.05). The principal gradient scores of left posterior cingulate gyrus are negatively correlated with the scores of CESD and HAMD(*P* < 0.05).

Next, we visualized the brain subdivisions that differed within the DMN based on an atlas of the Schaefer 7 network containing 200 Parcels (Fig. 7). Specifically, the differed brain subdivisions was located in the right posterior cingulate gyrus, the right precuneus, the left lateral superior temporal gyrus, the left middle temporal gyrus, the left anterior cingulate gyrus and the left posterior cingulate gyrus. Further correlation study showed that the gradient scores for left anterior cingulate gyrus in StD group were negatively correlated with the scores of HAMD (*P* < 0.05). Similarly, the gradient scores for left posterior cingulate gyrus in StD group were negatively correlated with the scores of CESD and HAMD (*P* < 0.05) (Fig. 8).

## 4 Discussion

Hierarchy is a key organizing principle of the human brain's functional networks, and it is unclear whether and how it changes in patients with StD. Our findings showed that patients with StD exhibited an altered overall hierarchy of the brain's functional networks, characterized by a contraction in the range of the two extremes (sensorimotor and DMN) and a suppression of the gradient in the cortical range, as compared to healthy controls. At the functional network level, reduced gradient scores of DMN were found in patients with StD compared to controls. Further correlation analyses revealed that DMN gradient scores in patients with StD were negatively correlated with the scores of CESD and HAMD. After identifying significant changes in the DMN gradient, we proceeded to explore changes in subdivisions within the DMN. Our study found that, compared to healthy controls, gradient scores of right posterior cingulate gyrus, right precuneus, left lateral superior temporal gyrus, left middle temporal gyrus, left anterior cingulate gyrus, left posterior cingulate gyrus were reduced in patients with StD. In addition, the gradient scores of left anterior cingulate gyrus were negatively correlated with scores of HAMD, while the gradient scores of left posterior cingulate gyrus were negatively correlated with the scores of CESD and HAMD.

Our study found that both groups showed a gradual axis of connectivity variations, spanning from a low-level sensorimotor network to a cross-modal DMN, with intermediate network being located in middle position. However, overall hierarchical pattern of patients with StD was altered, with increased primary cortices gradient scores in the sensorimotor and visual networks and reduced gradient scores in the limbic, frontoparietal control and default mode networks compared to healthy controls. This led to a contraction in the cortical gradients. According to previous studies, the primary cortex, such as the sensorimotor network, is furthest from the higher DMN, suggesting that neural activity in the DMN region may be relatively isolated from direct environmental input (Margulies et al., 2016). The longest physical distance from the primary cortex and DMN ensures a complete processing route from concrete stimuli to the integration of abstract concepts to facilitate higher brain functions (Smallwood et al., 2021). In addition to core depressive symptoms like prolonged depression, patients with StD also suffer from a number of higher brain dysfunctions such as poor concentration, sleep disturbances and reduced social skills (Crockett et al., 2020, Zhang et al., 2018). The abnormal contraction of the principal gradient axis in the brain of patients with StD indicates that the functional separation between functional networks in the brain is weakened, and that the functional integration and separation between lower and higher networks is disrupted. These changes may lead to abnormal information transition processes from sensory processing areas to higher areas, which in turn further exacerbates higher brain dysfunction in patients with StD.

At the functional network level, we found that the gradient scores of DMN were significantly lower in patients with StD compared to controls. Further correlational analyses found that the gradient scores of DMN in patients with StD were negatively correlated with the scores of CESD and HAMD, i.e. lower gradient scores reflected more severe depressive symptoms. Damage to the DMN which is involved in self-perception, emotion processing and monitoring of information from the internal and external environment, may be closely related to typical clinical manifestations of depression, such as depressed mood, impaired self-perception and ruminative thinking (Mulders et al., 2015, Zhong et al., 2016, Yan et al., 2019). Vulser et al. found that white matter microstructures within the DMN were altered in adolescents with StD compared to controls, and that these alterations were predictive index for future depression (Vulser et al., 2018). A recent resting state-fMRI-based radiomics study found that the amplitude of low-frequency fluctuation features within the DMN had a high discriminatory power in distinguishing between StD, MDD, and healthy controls (Zhang et al., 2022). Previous research indicated there was a correlation between negative emotional impact and the efficiency of DMN, as well as a correlation between negative emotional impact and subthreshold depressive symptoms. Specifically, DMN efficiency at rest is positively correlated with depressive symptoms and risk (Provenzano et al., 2021). Another study investigated how subthreshold depression symptoms affected DMN functional connectivity during the transition period of adolescence, and the results showed that, more severe depressive symptoms were associated with significant decreases in connectivity over time, indicating that StD symptoms alter the trajectory of DMN connectivity (Son et al., 2023). Another study, which used independent component analysis to investigate the functional connectivity changes of the resting DMN in two different age groups of StD patients, showed a significant increase in functional connectivity(FC) between DMN and the ventral striatum in StD patients compared to the control group. In addition, there was a significant positive correlation between FC and CESD scores between DMN and ventral striatum (Hwang et al., 2016). Consistent with those findings, we found that the alterations in gradient hierarchy were concentrated in the DMN, which provided further evidence for the significant role of the DMN in patients with StD.

Furthermore, the study explored the possible alterations of DMN in patients with StD. Compared to controls, the gradient scores in the DMN were significantly lower in the cingulate gyrus, precuneus and superior temporal gyrus, and the reductions in the parcel gradient scores in the anterior and posterior cingulate gyrus were associated with more severe depressive symptoms. The anterior cingulate gyrus is part of the limbic system of the brain, and serves as the bridge between attention and emotion processing, playing an important role in integrating visceral, attentional and emotional information (Zheng et al., 2018). Vulser et al. found a significantly reduced grey matter volume in the right anterior cingulate gyrus in 14-year-old adolescents with StD and an increased risk of having depression (Vulser et al., 2015). Phillippi et al. found that severity of depression in patients with StD was associated with reduced connectivity between pregenual anterior cingulate cortex (pgACC) and the striatum, as well as reduced connectivity between anterior subgenual anterior cingulate cortex (sgACC) and the anterior insula (Philippi et al., 2015). The posterior cingulate, a key part of the DMN, is a critical hub for self-referential processing, cognitive control and emotion processing, and serves as a cortex underlying multidomain cognitive function through its connections to distal cortical areas (Li et al., 2022). Dotson et al. found that patients with StD exhibited reduced cortical thickness in the right posterior cingulate gyrus, compared to the left posterior cingulate gyrus, and this change was associated with more severe somatic symptoms of depression (Dotson et al., 2021). In the present study, we found that the gradient subdivisions of the anterior and posterior cingulate gyrus within the DMN were significantly reduced in patients with StD, and this change was correlated with the severity of depression, which might be an important part of the pathological changes in patients with StD. The findings of this study are expected to be new biomarkers for the diagnosis and prognosis of patients with StD.

There were some limitations to this study. Firstly, the sample size of the study was relatively small, which may affect the statistical power and the accuracy of the results. Secondly, this study focused on young people with StD, but StD was also prevalent in other age groups such as older adults, and there was some heterogeneity in the results of the gradient analysis across age groups in healthy people. Therefore, it is unclear whether the same results would be observed in other age groups with StD. Future studies may consider a more comprehensive age range for gradient analysis of the StD population to obtain more comprehensive and accurate results. Finally, due to the limited behavioral information collected in this study, the impact of cortical range gradient scores on cognition has not been explored. Future research will further explore the relationship between gradient scores and cognition.

## 5 Conclusion

In summary, the study found that macroscale network hierarchy was altered in patients with StD and that such alterations were closely associated with depressive performance in patients with StD. These results may provide a new perspective for further research into the neural mechanisms of StD.

## Funding

This study was supported by the National Natural Science Foundation of China (Number 81804164), 2022 Research Project of Chinese Association of Rehabilitation Medicine (KFKT-2022-013), 2022 Major Scientific and Technological Innovation Project of Fujian University of Traditional Chinese Medicine (XJB2022006), the Youth Science and Technology Innovation Cultivation Program by Fujian University of Traditional Chinese Medicine (XQC2023003, XQC2023004).

## CRediT authorship contribution statement

**Xiaolong Yin:** Writing – review & editing, Writing – original draft, Methodology, Conceptualization. **Junchao Yang:** Writing – review & editing, Writing – original draft, Formal analysis, Conceptualization. **Qing Xiang:** Writing – review & editing, Software, Investigation, Data curation. **Lixin Peng:** Writing – review & editing, Resources, Methodology. **Jian Song:** Software, Resources, Data curation. **Shengxiang Liang:** Writing – review & editing, Visualization, Validation, Supervision, Project administration. **Jingsong Wu:** Validation, Supervision, Resources, Funding acquisition.

## Declaration of Competing Interest

The authors declare that they have no known competing financial interests or personal relationships that could have appeared to influence the work reported in this paper.

## Data Availability

Data will be made available on request.
